# Typhoid Fever and Typhoid Carriers

**Published:** 1908-03

**Authors:** D. S. Davies, I. Walker Hall

**Affiliations:** Medical Officer of Health, Bristol; Professor of Pathology, University College, Bristol; Pathologist to the Royal Infirmary


### TYPHOID FEVER AND TYPHOID CARRIERS.

BY

D. S. Davies, M.D.,

*Medical Officer of Health, Bristol,*

AND

I. Walker Hall, M.D.,

*Professor of Pathology, University College, Bristol;*
*Pathologist to the Royal Infirmary.*

---

Typhoid fever has shown in England and Wales, during the three and a half decades that have elapsed since it was distinguished in the Registrar-General's returns from typhus fever, a continuously decreasing *mortality* :—

| Periods. | Death Rate per Million Living. |
|---|---|
| 1871—1880 | 322 |
| 1881—1890 | 198 |
| 1891—1900 | 174 |
| 1901—1905 | 110 |

While the *mortality* has decreased, however, the *fatality* (or case-mortality) has remained practically stationary. Thus, in the London Fever Hospital in 1848—1857 the fatality rate was 17·3 per cent., in the Metropolitan Asylums Board Hospitals in 1871—1897 the rate was again 17.3 per cent., and in the single year 1897 it was 18.7 per cent.

Upon the discovery of the bacillus of typhoid fever in the eighties, it was anticipated that control of the disease would become simple ; the causal bacilli were supposed to enter, occupy, and leave the body as an orderly army, leaving no stragglers, and recovery of the patient was held invariably to connote the death and disappearance of the bacilli.

If this were always so, the control of such a disease would be comparatively simple, and the knowledge that the typhoid organism is a flagellate water-living parasite leaving the body by way of the solid or liquid excretions during the clinical continuance of the disease, and completing its life history by a more or less prolonged saprophytic existence in organically polluted soil, or in water, pending its introduction into the intestinal tract of its next host, would sufficiently explain the etiology of the disease in its well-known phases as water-borne, milk-borne, oyster-carried, and so on.

But allowing for the fulfilment of these conditions in the majority of instances, there still occurred a residuum of cases which did not fall into line with these routine conditions, and fuller knowledge of the life history of the causal parasite has become necessary in order to account for such aberrant outbreaks.

That the major portion of the decline in the prevalence of typhoid fever has been due to general improvement in drainage and water supply is probably, in view of the nature of the disease, incontrovertible, and this obvious relationship has tended to obscure the validity of personal transference (mediate or immediate) of infection.

The easy habit has unfortunately arisen of referring obscure sporadic outbreaks of typhoid fever to general insanitary conditions, and the smoke test has no doubt led to the reconstruction of much innocent drainage, and to an outlay which might have been better expended.

Two things, however, became in the course of time apparent: one that, owing to enhanced resistance to infection in certain individuals, attacks in such partly-immune persons might be represented by very slight or negligible symptoms, so that neither the patient nor his friends, nor even his medical attendant would have reason to suspect the real nature of his illness. Hence arise "ambulant" cases and "missed" cases, affording far greater opportunities for the distribution of infection owing to ignorance of any need for, and consequent lack of, precautions; the other, that after apparent recovery, some of the bacterial diseases may persist in the patient, who, himself immune, may become a "carrier" of the disease, and be potentially infective towards others.

The occasional persistence of the Bacillus Typhosus in the human body over long periods has been recognised for some years, and the possibility of this condition forming the explanation of obscure outbreaks was suggested by Horton Smith in his Goulstonian Lectures in 1900. At that time it was generally accepted that the Bacillus Typhosus could be demonstrated in the stools, if care was taken, during the first and second and early part of the third week of the disease, and possibly also during the early part of the relapse. Later, it could not be demonstrated by the laboratory methods then available.

It was known, however, from the experiments of Chiari[1] that the bile in the gall-bladder contains the typhoid bacillus in the majority of cases, and often in pure culture.

It was naturally supposed at first that, as the fever passed away, and as health returned, the bacillus, the cause of the disease, would disappear from the tissues also, and this was demonstrated to be the case in some of the earliest observations.

But the experiments of Blackstein and Welch[2] showed that, sometimes at least, the typhoid bacillus might remain for much longer periods; experimentally, in rabbits, it was found in the bile 128 days after the date of inoculation, and this suggested that the same thing might occur in man.

Between 1894 and 1896, Buschke[3], Sultan[4], and Bruni[5] quoted cases in which the bacilli were found in the pus from bone abscesses, in two instances six years after the primary fever, and in one case seven years after.

Hunner[6] quotes cases in which cholecystitis was shown to be accompanied by the presence of the Bacillus Typhosus in pure

[1] *Ztschr. f. Heilk.*, 1894, xv. 199.
[2] *Johns Hopkins Hosp. Bull.*, 1891, ii. 96, 121.
[3] *Fortschr. d. Med.*, 1894, p. 572.
[4] *Deutsche med. Wchnschr.*, 1894, p. 34.
[5] *Ann. de l'Inst. Pasteur*, 1896, x. 220.
[6] *Arb. a. d. k. Gsndhtsamte.*, 1895, p. 207.

culture; in one case three months after the fever, in another eight months, and in a third after an interval of seven years. The most remarkable case of all, however, and one very carefully tested, is that recorded by Von Dungern[1], in which fourteen and a half years after the attack of typhoid fever the bacilli were still present in pure culture in the cystic contents.

These facts, detailed by Horton Smith, and insisted upon by him as possible sources of re-infection, have remained largely ignored in their epidemiological relationship, partly because the assumed rarity of their occurrence suggested that they were merely bacteriological curiosities, and partly because the isolation of the Bacillus Typhosus from the fæces was at that time not only difficult, but very uncertain.

In 1906, however, owing to the continued prevalence of sporadic typhoid in South Germany, the subject was taken up with the advantage of modern and more certain laboratory methods, and Klinger[2] found that persons in apparent health can harbour typhoid bacilli and excrete them in the stools or urine.

The German observers divide "carriers" into two classes:—

1. "Acute carriers," who have shown no symptoms, but after being in direct contact with patients may carry and excrete bacilli for a short time and in small numbers.

2. "Chronic carriers," who have a short, or a long, time before gone through a regular attack of typhoid, and may excrete for months, or years, more or less pure cultures of typhoid bacilli.

The "chronic carriers" are obviously the most dangerous class. About 4 per cent. of typhoid patients appear to become carriers; the condition is most common in women, and the bacilli, in all probability, are harboured in the bile in the gall-bladder, or even in the intra-hepatic biliary passages, or hepatic focal necroses, whence intermittently they are discharged and excreted with the fæces. This condition may persist for as long as ten years, possibly longer. When these chronic carriers are engaged in the preparation of food, or in dairy work, they are apt to give rise to intermittent local outbreaks of typhoid fever,

[1] *München. med. Wchnschr.*, 1897, xliv. 699.

[2] *Arb. a. d. k. Gsndhtsamte.*, 1906, xxiv. 91.

probably by contamination of the food with the hand after defæcation or micturition.

Kayser[1] quotes the case of a proprietress of a Strasburg bakehouse who prepared the meals for the employés, and states that each journeyman developed typhoid soon after arrival. Examination of her stools yielded cultures of typhoid bacilli, and her blood gave a distinct Widal reaction (1—100).

Soper,[2] of New York, records the case of a cook who in five years lived in four families, and gave rise to twenty-eight cases. The bowel discharges furnished practically pure cultures of the typhoid bacillus; the extrusion of bacilli was intermittent.

A. and J. C. G. Ledingham[3] record thirty-one cases in fourteen years at an institution, finally traced to carrier cases.

At a home for inebriates near Bristol, containing 240 inmates, a succession of twenty-eight cases of typhoid fever occurred intermittently between November, 1906, and November, 1907, and caused two deaths. The introduction of the first case could not be explained, but transference of the infection was obviously by milk, which, moreover, became infected *after* sterilisation.

It was supposed that rats, having access to the dairy and food stores from the drains, conveyed the infection, but when these premises were made rat proof the outbreak still went on.

Finally, suspicion fell upon a woman employed in the kitchen and dairy work during the whole time of the outbreak, who had suffered from a severe attack of typhoid fever in 1901. She was excluded from kitchen and dairy work and isolated, and no further cases occurred except one, twelve days after her isolation commenced, and so within the incubation period.

This patient was apparently in perfect health; her blood agglutinated a laboratory strain of bacillus typhosus in low dilutions, and her fæces, when examined on two occasions in November, 1907, appeared to be perfectly normal. But on December 20th, after slight abdominal pain, she passed a loose light stool, not unlike an early typhoid stool, and from this stool typhoid bacilli were recovered in considerable quantities.

[1] *Ibid.*, 176. [2] *Med. Rec.*, 1907, lxxvi. 818.
[3] *Brit. M. J.*, 1908, i. 15.

It was subsequently discovered that this same woman had been cook at an institution for children at a time when, in 1904, twenty-five out of the fifty inmates developed typhoid fever at intervals within five months.

The cases quoted sufficiently indicate the "efficiency" of a "typhoid carrier" as a source of infection, and forcibly suggest the necessity, in any outbreak, for inquiring into "personal" as well as "local" possibilities of infection.

The recorded outbreaks present many points of similarity; the cases occur in groups at uncertain intervals, for the bacilli are extruded intermittently. The effective carrier is usually, or always, employed in food preparation; if she also handles the milk supply the opportunities for infection are greatest, and the percentage of attacks is large. Institutions where a number of persons spend a considerable time, and receive food which passes through the same person's hands, offer the most striking and most easily studied examples of this method of infection.

*Prophylaxis.*—The stated percentage (4 per cent.) of convalescents who become carriers is considered by Savage[1] and others to be probably understated; but it must be admitted that only a few seem to achieve distinction, which may be due to variation in habits of cleanliness.

It would be difficult to enforce the rule that every typhoid convalescent should prove the dejecta to be free from the Bacillus Typhosus for three months, before being regarded as other than a suspected "carrier." Still, such a precaution would probably minimise the occurrence of sporadic outbreaks, and the resulting saving in sickness and mortality would repay the tedious routine.

In any case, the admission of typhoid convalescents to institutions and private homes must be considered seriously.

Stringent regulations can readily be made and enforced, with proper discipline, in institutions, especially with regard to the absolute necessity for the adequate cleansing of the hands after micturition and defæcation, of all persons engaged in kitchen or dairy work, carriers or not, before handling food; and care

[1] Savage, "Recent Work upon the Bacteriology of Typhoid Fever," *Pub. Health*, 1907, xx. 12.

should certainly be taken, by inquiry into the history of past illnesses, to avoid the appointment of persons who may be "carriers" of typhoid to the position of cook or dairymaid.

If persons are known to be "carriers" they must, of course, be excluded from all dealings with food preparation, and frequent changing and disinfection of the underlinen, care in the cleansing of the hands. and other obvious precautions should be enforced.

*Treatment.*—From the standpoint of treatment the "chronic carrier" provides an intricate problem. To remove the typhoid bacilli from the intestine does not appear to be a difficult task when the lists of purgatives learned in one's earlier days come up for revision. But how to dislodge them from the gall-bladder or the liver is, as yet, an unsolved question. The surgeon may remove the gall-bladder, when permitted, but even he would scarcely dare to excise the liver. The failure of the ordinary intestinal antiseptics in these cases is a pronounced one, and the result of experiment must be waited for in patience.

It is possible that the treatment of the convalescent stage of typhoid fever calls for newer methods and must undergo revision; if drugs fail, bacterial agencies may in time be found to secure the elimination of the Bacillus Typhosus from the body.

It is at any rate certain that typhoid convalescents cannot be lightly discharged as cured because the clinical manifestations have ceased, and that some measure of subsequent observation and control should become a part of routine treatment.

---

